# Silicon Effects Depend upon Insect Herbivore Guild and Has Limited Influence on Gall-Inducing Insects of *Bauhinia brevipes*

**DOI:** 10.3390/plants14020250

**Published:** 2025-01-17

**Authors:** Guilherme Ramos Demetrio, Henrique Venâncio, Janaina Correa Batista, Jean Carlos Santos

**Affiliations:** 1 Laboratory of Plant Ecology, U.E. Penedo, Campus Arapiraca, Federal University of Alagoas, Penedo 57200-000, Brazil; guilherme.ferreira@penedo.ufal.br; 2 Programa de Pós-Graduação em Ecologia & Conservação, Universidade Federal de Sergipe, São Cristóvão 49107-230, Brazil; henrivens@gmail.com; 3 Instituto de Biologia, Universidade Federal de Uberlândia, Uberlândia, Minas Gerais 38405-320, Brazil; janainabatistacorreia@gmail.com; 4 Departamento de Ecologia, Universidade Federal de Sergipe, São Cristóvão 49107-230, Brazil

**Keywords:** plant defense mechanisms, endophytic herbivores, induced defense, plant–herbivore interactions, plant resistance

## Abstract

Silicon (Si) is a widely recognized element in plant defense, often enhancing resistance to herbivory by strengthening cell walls and deterring feeding by external herbivores. However, its impact on internal, endophytic herbivores, such as gall-inducing insects, remains underexplored. This study investigates the role of silicon in *Bauhinia brevipes*, focusing on its effects on herbivory by insects. We hypothesize that while silicon strengthens plant tissues and reduces feeding by external herbivores, it may have a limited effect on internal feeders, such as gall-inducing insects. Our results indicate that silicon accumulation in leaves significantly reduces herbivory by chewing insects but has no direct effect on the occurrence of gall-inducing insects. Silicon content in galled tissues was lower compared to healthy leaves, suggesting that gall-inducing insects may manipulate silicon distribution to mitigate its defensive effects. Our results indicate that hypersensitivity reactions were positively influenced by silicon, highlighting the role of this element in enhancing localized defense mechanisms. Our findings reveal silicon’s tissue-specific roles in plant defense, emphasizing the need for more research on its nuanced interactions with endophytic herbivores and implications for ecological applications. This research contributes to the literature on silicon’s multifaceted role in plant–herbivore interactions and its potential applications in sustainable pest management.

## 1. Introduction

Herbivory is a central ecological interaction that has driven the evolution of complex plant defense mechanisms [1]. Plants face continuous pressure from herbivores and have developed a variety of defensive strategies to mitigate the impact of herbivory, ranging from structural barriers to chemical deterrents [2]. These adaptations are particularly dynamic in tropical ecosystems, where the diversity and abundance of herbivores increase the selection pressures on plants, promoting intricate defenses and even inducible responses to deter their herbivores [3,4]. Thus, the understanding of these interactions is essential for elucidating the ecological and evolutionary significance of plant defenses in natural ecosystems.

Plants have developed a diverse array of defense mechanisms to mitigate and/or avoid herbivory. Physical defenses, such as trichomes and cell wall thickening, are often coupled with chemical defenses, including compounds like phenolics and alkaloids, to reduce palatability and slow herbivore growth [5]. In this context, the hypersensitivity response (HR) has emerged as an effective defense against insect herbivores, providing a localized reaction that limits damage and contains nutrient loss [6]. While HR was traditionally associated with pathogen resistance, studies now suggest it also serves as an herbivore deterrent, particularly against insects, by inducing cell death around the feeding site, thereby restricting further damage [7,8]. Together, these defenses create a multifaceted system that enables plants to withstand and adjust to herbivory pressure.

Silicon (Si) has gained attention as a key element in plant defense against environmental stresses, including herbivory [9,10]. Silicon has been identified as a part of multimodal defense mechanisms, which effects range from higher attractiveness of natural enemies [11] to differential protein expression, presenting direct and indirect effects on herbivores and herbivory defense mechanisms [12]. Silicon accumulates in plant tissues, enhancing structural rigidity and deterring herbivory by making leaves tougher and less digestible [13,14]. This mineral element has been shown to reduce herbivore feeding rates and overall damage, establishing its protective role, particularly in environments with high herbivory [15]. A recent meta-analysis has shown that silicon-rich plants experience herbivory levels ~33% lower than silicon-poor plants [16]. In addition, silicon’s role in herbivory resistance can be amplified when combined with other defense mechanisms, providing a comprehensive approach to deterring herbivores through multiple channels [17].

Besides its well-established importance on herbivory defense, recent studies have proposed that the feeding habit of the herbivore can be a determinant for its sensitivity to Si-induced defenses [16]. The effects of Si defenses against exophagous herbivores are well established in the literature, and the attacks of feeding guilds like phloem-feeders [18] and chewers [15] are commonly referred to as being lower in plants with higher Si content. In this context, fluid-feeders appear to be less adversely affected by Si-induced defenses when compared to chewing insects, mammals, and plant-boring arthropods [16]. However, Si effects on the resistance against endophytic herbivores are less clear and overlooked in herbivory defense studies.

**Figure 1:**
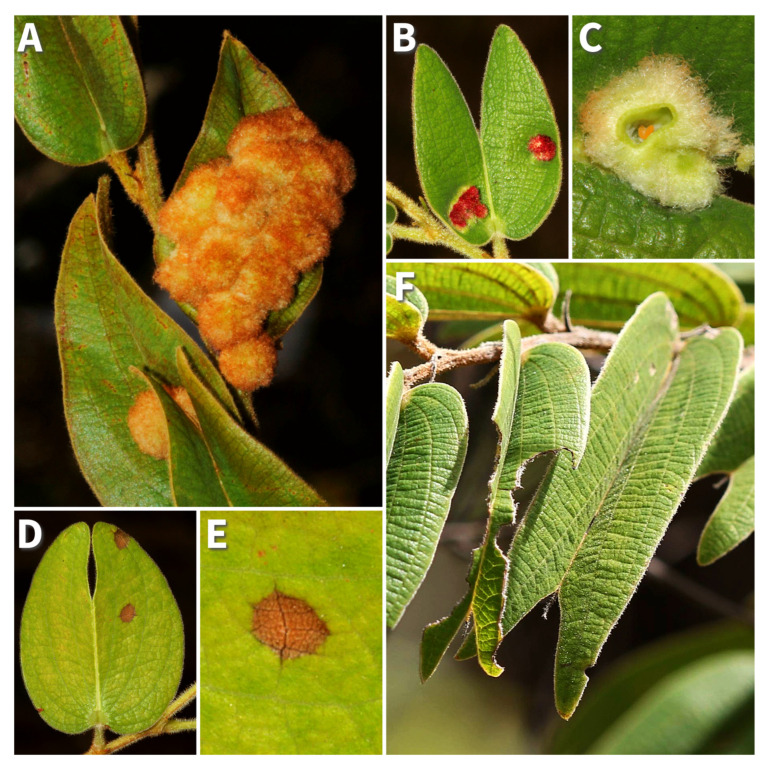
*Bauhinia brevipes* system: (**A**) mature and (**B**) young galls of *Schizomyia macrocapillata* Maia, 2005 (Diptera: Cecidomyiidae); (**C**) a cross-sectional view detailing the inducer (orange larvae) in the gall center; (**D**) leaf exhibiting hypersensitivity reactions (HRs); (**E**) HR in detail; and (**F**) leaf displaying herbivory by chewing insects juxtaposed with an undamaged leaf.

The *Bauhinia* genus represents a focal group for studies on plant–herbivore interactions due to its complex relationships with insect herbivores and gall-inducing insects, as well as its array of defensive strategies [19,20]. Known for its structural defenses [21], *Bauhinia* also demonstrates inducible responses to herbivory as HRs (hypersensitivity reactions), and that may include silicon accumulation [8]. Studies on *Bauhinia brevipes* Vogel revealed how this plant leverages both silicon and HR as effective responses against insect herbivores and gall-forming insects, which are known to specialize in attacking determined plant species and tissues [22]. In this host plant, HR is better understood in the defense against galls of *Schizomyia macrocapillata* Maia, 2005 (Diptera: Cecidomyiidae) [8,23,24]. HR is induced during the early ontogenetic stages of *S. macrocapillata* galls, resulting in almost 90% mortality of this gall-inducing population [23]. However, little is known about the relationship between HR and other defense mechanisms in the *B. brevipes* system (Figure 1). Therefore, exploring the dual role of silicon and HR in *Bauhinia* provides valuable insights into the adaptive significance of these defenses against highly specialized insect threats.

The present study investigates the connections between silicon and the occurrence of gall-inducing insects, with a focus on hypersensitivity reactions as potential defense mechanisms, in *B. brevipes*. Specifically, we aim to determine whether silicon accumulation in leaves of *B. brevipes* correlates with a decrease in gall formation and if HR modulates this relationship by containing damage locally. To address these questions, we examined silicon content across different leaf tissue types: galled, ungalled, and gall tissue. Thus, our aim was to understand the relationship between the foliar Si content of *B. brevipes* and herbivory by chewing insects and gall-inducing insects. We sought to answer the following questions: 1. Does the amount of silicon in leaves affect the occurrence of gall-inducing insects in *B. brevipes*? 2. Does the amount of silicon in leaves mediate defense mechanisms against chewing and gall-inducing herbivores in *B. brevipes*? and 3. Is there a difference in silicon content when comparing tissues of leaves without galls, leaves with galls, and the gall tissue itself? We hypothesized the following: 1. The higher the silicon content, the lower the number of galls found on the leaves of *B. brevipes*; 2. Silicon has a negative effect on the attack rates of chewing insects; 3. Leaves with galls will have lower silicon content compared to leaves without galls, and the silicon content in the gall tissue will be lower than in the adjacent tissue of gall-bearing leaves, due to the ability of galling insects to manipulate chemical compounds in plant tissues.

## 2. Results

Gall numbers per plant varied from 0 to 52, with an average of 4 ± 11 galls per plant. The average herbivory rate was 2.57 ± 3.45%, ranging from 0 to 15.97%. The mean silicon content in leaves was 0.13 ± 0.02, varying between 0.09 and 0.17. The mean defense rate was 86.47 ± 23.19%, with values ranging from 0 to 100%. The number of hypersensitivity reactions (HRs) averaged 36.6 ± 42.44, with a range from 0 to 257.

**Figure 2:**
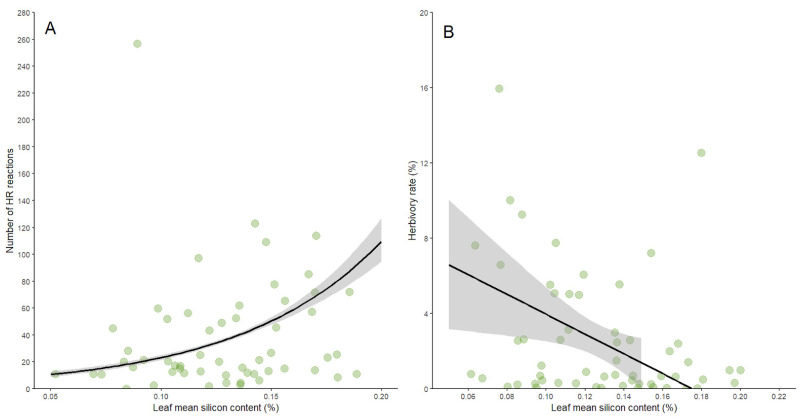
Scatter plots showing the positive relationship between silicon content and the number of hypersensitivity reactions (HRs) (**A**) and the negative relationship between silicon content and the herbivory rate (**B**) in *Bauhinia brevipes* leaves. The green circles represent each observation, and the black line represents the fitted model. The shaded areas represent the confidence intervals. The GLM fit for both models is shown with statistical significance indicated (*p* < 0.05 for HR, *p* < 0.01 for herbivory rate).

The number of galls per plant was not influenced by the mean silicon content in leaves (d.f. = 1, *p* = 0.723), suggesting that gall-inducing insects may bypass silicon-based defenses. Regarding defense mechanisms, although the defense rate of *B. brevipes* was not related to the mean silicon content in leaves (d.f. = 1, F = 0.789, *p* = 0.378), the number of HRs was positively influenced by silicon content (R^2^ = 0.97, Table 1, Figure 2A). Conversely, herbivory by chewing herbivores was negatively influenced by silicon content in leaves (R^2^ = 0.09, Table 1, Figure 2B), indicating differential effects on plant defenses.

**Figure 3:**
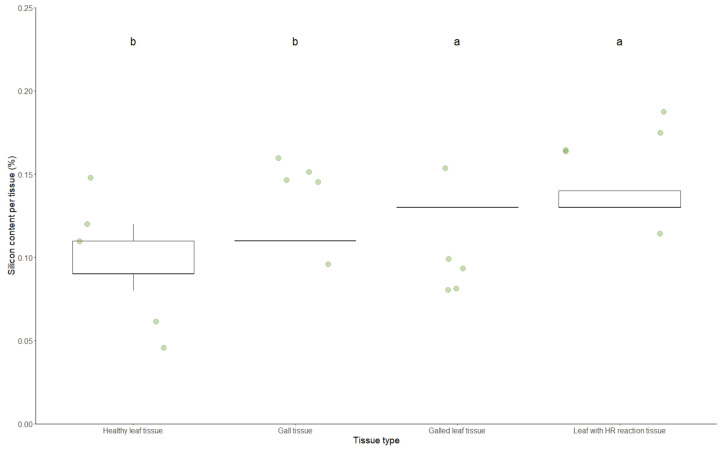
Box plots illustrating the distribution of silicon content in different leaf tissue types: healthy, galled, with hypersensitivity reactions, and gall tissue. The green circles represent each observation, and the boxes represent 50% of the data, from the first to the third quartile. The statistical differences between tissue types were assessed using GLMs with a Gaussian distribution and are marked accordingly. Different letters over the boxes indicate statistically significant differences in the silicon content per tissue (%).

Silicon content varied among different tissue types (d.f. = 3, F = 11.11, *p* = 0.00003). Gall-bearing leaves and leaves with hypersensitivity reactions did not differ significantly in silicon content and exhibited the highest values. Both tissue types differed from gall tissues and healthy leaves, which did not differ from each other and displayed the lowest silicon content (Table 2 and Figure 3).

**Figure 4:**
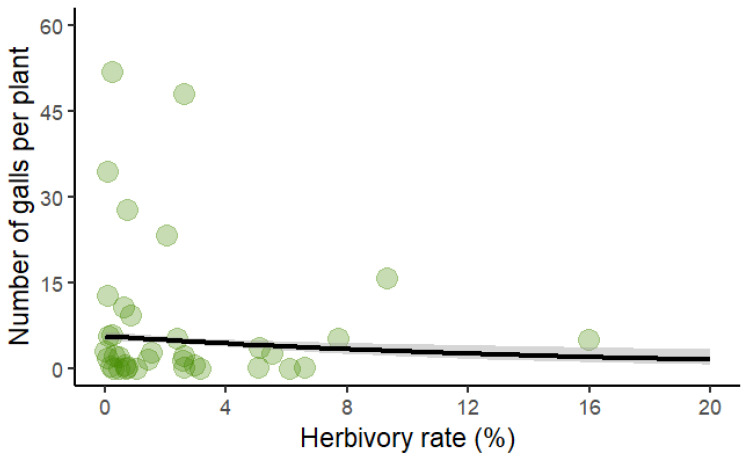
Scatter plot showing the negative correlation between herbivory rate and the number of galls per leaf in *Bauhinia brevipes*. The green circles represent each observation, and the black line represents the fitted model. The model fit is based on a Poisson-distributed GLM, with statistical significance indicated (*p* < 0.01). The shaded area represents the confidence interval.

Finally, the number of galls was negatively affected by the herbivory rate, with more heavily consumed leaves exhibiting a lower number of galls (Table 3, Figure 4).

## 3. Discussion

Our study provides new insights into the silicon (Si) content and its potential connection with herbivore interactions in *Bauhinia brevipes*, revealing its differential impact on plant–herbivore interactions, aligned with prior studies [11,13,14,25,26,27,28,29,30]. The silicon did not directly influence the number of gall-inducing insects on the host plant, but our results suggest that it is strongly linked to hypersensitivity reactions (HRs), a localized defense that is crucial against specialized herbivores. Additionally, a strong negative correlation was observed between foliar silicon content and herbivory by chewing insects, reinforcing the protective role of silicon as a structural plant defense [16]. Intriguingly, we also found that galls exhibited lower silicon content compared to tissues associated with HRs and ungalled leaves, suggesting a potential mechanism by which gall-inducing insects may manipulate silicon distribution to mitigate its defensive effects through the galls [16,18].

Our findings underscore the multifaceted role of silicon in plant defense, particularly in ecosystems like the Cerrado, where plants are under intense herbivory pressure [31,32,33]. The complementary nature of silicon and HRs in *B. brevipes* reflects an adaptive advantage in the Cerrado, where high herbivore diversity exerts strong selective pressures [33]. While silicon alone may be insufficient to deter gall-inducing insects, its role in enhancing HR could provide a synergistic defense by increasing the mortality of gall-makers during early ontogeny [23]. In this case, the increased Si content in the leaves of *B. brevipes*, which were heavily infested with gall-inducing insects, resulted in reduced herbivory. We propose that the distribution of herbivorous insects on the host plant may be mediated by both constitutive (Si) and induced (HR) plant defense mechanisms, resulting in indirect interactions between herbivore insect guilds. Numerous studies have demonstrated that herbivores can interact directly or indirectly, mediated by the host plant (e.g., [34]).

Interestingly, the similar silicon content in galled leaves and healthy leaves, combined with the lower silicon content in gall tissues, allows us to hypothesize that gall-inducing insects may actively manipulate silicon allocation within their host. Previous studies have established that galls can accumulate secondary metabolites produced by plants to improve their own survival [35,36] and also to control the nutrient levels in the gall tissue in comparison with the surroundings of the leaf [37]. In this sense, silicon uptake can be linked to herbivory intensity, and more damaged leaves seem to accumulate higher rates of this element [38]. Therefore, by mobilizing silicon away from the attacked tissues, gall-makers might reduce its defensive effects and create a more favorable microenvironment for their development. This hypothesis is supported by studies suggesting that gall-inducing insects can hijack host resources to suppress plant defenses and sustain gall growth [17,19,39].

While our study provides compelling evidence for the defensive role of silicon, it is limited by the sample size of galled tissues and the reliance on observational data for some analyses. Experimental manipulations of silicon content in controlled environments could provide more definitive evidence of causality. Additionally, environmental factors such as soil silicon availability, water stress, and plant nutritional situation, which were not explicitly measured, might influence the observed patterns [40,41]. The apparent redistribution of silicon in galled tissues could also reflect a passive response to gall formation rather than active manipulation by gall-makers, a hypothesis requiring further investigation.

The role of silicon in enhancing plant defense has practical implications for both natural and managed ecosystems. Silicon supplementation in agricultural settings has been shown to reduce herbivore damage and improve crop resilience [9,15,42]. The findings of our study suggest that similar strategies could be applied in biodiversity-rich systems like the Cerrado to bolster plant defenses against herbivory. Additionally, understanding how gall-inducing insects manipulate silicon distribution could inform pest management strategies that disrupt such mechanisms, reducing the success of these specialized herbivores.

Future research should explore the biochemical and molecular mechanisms underlying the interaction between silicon and HR, particularly how silicon may prime or amplify HRs. Studies investigating the genetic and physiological pathways involved in silicon redistribution in galled tissues could shed light on the ability of gall-inducing insects to manipulate host defenses. Comparative analyses across other *Bauhinia* species and silicon-accumulating plants would help clarify whether these patterns are species-specific or represent a broader ecological strategy. Finally, the inclusion of soil and environmental variables in experimental designs would provide valuable context for understanding the conditions that modulate silicon-mediated defenses.

## 4. Materials and Methods

### 4.1. Study Area

Our study occurred at the Panga Ecological Station (EEP) (19°10′55″ S–48°23′35″ W), located in Uberlândia, Minas Gerais, Brazil. The EEP is a private natural reserve owned by the Federal University of Uberlândia and encompasses various Cerrado vegetation formations, including grassland, savanna, and forested areas [43]. The soil is hydromorphic latosol with a sandy texture and acidic properties [44]. The altitude ranges from 750 to 830 m [44], and the climate is Aw type, according to Köppen’s classification, characterized by rainy summers (October to March) and dry winters (April to September). The average annual temperature and total precipitation are 22 °C and 1500 mm, respectively.

### 4.2. Field Sampling

We sampled *B. brevipes* individuals from December 2014 to February 2015, during the rainy season. The rainy period coincides with both the sprouting of new leaves and the peak of gall-maker attacks, including *S. macrocapillata* [45]. To avoid heterogeneity in anti-herbivory responses [46], we sampled plants under similar environmental conditions (i.e., sunny areas and located in the same soil type). Additionally, we selected only individuals that showed signs of herbivory by chewer insects, gall-inducers, and/or with hypersensitivity reaction (HR) marks. Damage of other herbivore guilds (e.g., sap suckers and scrapers) was rare in the evaluated *B. brevipes* population. Overall, we included 58 *B. brevipes* individuals in this study.

### 4.3. Foliar Herbivory

To evaluate the variables of herbivory and defense in *B. brevipes*, we randomly collected 30 fully expanded leaves from each of the 58 individuals (n = 1200 leaves). These leaves were identified and transported fresh in a thermal bag to the laboratory, where they were scanned at 300 dpi in a flatbed scanner (HP^®^ LaserJet Pro MFP M127fn, HP, Uberlândia, Brazil). Subsequently, we used digital images to estimate the percentage of leaf area consumed by chewing insects for each plant [22], calculated as the mean proportion of area removed by chewers (cm^2^) and total leaf area (cm^2^). We also analyzed the incidence of *S. macrocapillata* on all sampled leaves. For this, each gall was opened, and the number of gall inducers was counted based on the number of larval chambers. This method ensured data sampling accuracy, as the galls of this species are unilocular but occur in clusters on leaves [47]. Gall-inducing species were identified based on gall color, shape, and trichome density, as described in [48]. Additionally, we counted the number of HR marks, considering their color, shape, and position: brown circular marks on the adaxial surface of the leaves [49].

### 4.4. Quantification of Foliar Silicon Content

To evaluate whether foliar Si concentration is related to leaf damage caused by chewing and gall-inducing insects, we analyzed the silicon content in four sets of leaves: (1) 15 fullly healthy (i.e., without leaf damage caused by herbivores or pathogens) expanded leaves of 40 random plants (n = 600 leaves); (2) the same set of leaves to analyze herbivory by chewing insects (n = 600); (3) in a total of 50 leaves with *S. macrocapilata* galls, where we separated the galls from the leaf lamina and conducted the silicon analysis in both the gall tissue and the adjacent foliar tissue; and (4) in a total of 50 leaves with HR marks and no evidence of damage from herbivores or pathogens. We sampled only 50 leaves to evaluate the Si from galls and HR due to the low number of sampled plants of *B. brevipes* attacked by *S. macrocapilata* (10 plants).

We conducted the foliar silicon analyses at the Laboratório de Análise de Fertilizantes (LAFER) of the Institute of Agricultural Sciences at the Federal University of Uberlândia (UFU). All leaves were placed in an oven at an average temperature of 50 °C for 72 h to completely extract the water content, and then the leaves were ground using a Willey mill (Solab, Piracicaba, Brazil). Finally, we analyzed the foliar Si concentration following the methods proposed by [50].

### 4.5. Data Analysis

We used the ‘describe’ function from the “psych” [51] package to obtain descriptive statistics for our data. To test the effect of leaf silicon content on the occurrence of gall-inducing arthropods in *B. brevipes*, we constructed a generalized linear model (GLM) with a Poisson distribution, where the number of galls was set as the response variable and the mean silicon content in leaves was included as the predictor variable.

To evaluate whether silicon content mediates defense mechanisms against chewing herbivores and gall-inducing insects in *B. brevipes*, we built GLMs with a Gaussian distribution. In these models, the defense rate, the number of HR, and the individual herbivory rate were used as response variables, while the average silicon content in leaves was the predictor variable.

To test for variation in silicon content across different tissue types, we used a GLM with a Gaussian distribution, where silicon content in the tissue was the response variable and tissue type (gall-bearing leaf, healthy leaf, hypersensitivity reaction-bearing leaf, or gall tissue) was the predictor variable.

To assess the effect of silicon on the relationship between the number of galls and herbivory by chewing herbivores in *B. brevipes* individuals, we constructed a GLM with a Poisson distribution. In this model, the number of galls was the response variable, and the herbivory rate on leaves was the predictor variable.

Models were simplified to their most parsimonious forms based on Akaike information criterion (AIC). Diagnostic checks were conducted using residual plots to assess model fit, and dispersion parameters were examined to verify the appropriateness of the chosen distributions. For significant models, we calculated the R^2^ using the ‘rsquared’ function from the “piecewiseSEM” [52] package. All analyses were performed in the R environment (version 4.3.1) [53], and all graphs were generated using the ‘ggplot’ function from the “ggplot2” package [54].

## 5. Conclusions

This study highlights the complex role of silicon (Si) in plant defense, particularly in its differential impact on external versus internal herbivores. While Si effectively reduces herbivory by chewing insects through its biomechanical effects, it appears less effective against gall-inducing insects. This paradox suggests that endophytic herbivores, such as gall-inducing insects, may circumvent or manipulate the plant’s silicon defenses, potentially by redistributing silicon within the plant tissues. Furthermore, our findings suggest a strong association between silicon content and hypersensitivity reactions to gall-inducing insects, indicating a possible role for Si in modulating this localized defense mechanism, providing additional evidence for its multifaceted function in plant defense.

Our results contribute to the growing body of literature on silicon’s role in plant–herbivore interactions and suggest that its protective effects may be more nuanced than previously understood. While silicon may reinforce mechanical defenses, its impact on biochemical defenses and endophytic herbivores requires further exploration. Understanding how gall-inducing insects interact with silicon-rich plants could provide new and important insights into the adaptive strategies of both plants and insect herbivores. Future studies should focus on the molecular mechanisms underlying these interactions, including how silicon affects the redistribution of resources within plants and influences defense signaling pathways. Additionally, examining the role of silicon in other plant species and ecosystems could help to generalize our findings and refine its potential applications in integrated pest management.

## Figures and Tables

**Table 1:** Results from generalized linear models (GLMs) showing the relationship between mean silicon content in leaves and plant defense mechanisms in *Bauhinia brevipes*. The defense mechanisms measured include the defense rate (percentage of defensive tissue response), the number of hypersensitivity reactions (HRs), and the herbivory rate (% damage). Silicon content was found to positively influence HRs and negatively affect herbivory by chewing herbivores. No significant correlation was found between silicon and the overall defense rate. *p*-values were derived using GLMs with Gaussian distribution, and significant values (α = 0.05) are presented in bold.

Response Variable	Source of Variation	Estimate	Std. Error	t-Value	*p*-Value	R^2^
Number of HRs	Intercept	1.579	1.017	1.553	0.1261	-
	Mean silicon content	15.587	7.568	2.060	**0.0441**	0.97
Herbivory rate (%)	Intercept	9.220	2.772	3.326	**0.0015**	-
	Mean silicon content	−52.621	21.649	−2.431	**0.0183**	0.09

**Table 2:** Variation in silicon content among different leaf tissue types (healthy leaf, galled leaf, leaf with hypersensitivity reactions, and gall tissue). Galled and hypersensitivity reaction-bearing leaves showed significantly higher silicon content than gall tissues and healthy leaves. Significant differences in silicon content were assessed using GLMs with a Gaussian distribution, with a focus on tissue types as predictor variables. Significant *p*-values (α = 0.05) are presented in bold.

Comparison	Estimate	Std. Error	z-Value	*p*-Value
Gall versus healthy leaves	0.014	0.008	1.75	0.297
Gall-bearing leaves versus healthy leaves	0.04	0.008	5.00	**<0.001**
Leaves with HR versus healthy leaves	0.036	0.008	4.50	**<0.001**
Gall-bearing leaves versus hall	0.026	0.008	3.25	**<0.01**
Leaves with HR versus gall	0.022	0.008	2.75	**<0.05**

**Table 3:** Results from generalized linear models (GLM) showing the relationship between herbivory rate and the number of galls per leaf in Bauhinia brevipes. The data reveal that as herbivory increases, the number of galls decreases, suggesting a potential trade-off between herbivory and gall formation. The analysis was conducted using a Poisson distribution, where the herbivory rate was the predictor variable. Statistically significant *p*-values (α = 0.05) are presented in bold.

Source of Variation	Estimate	Std. Error	z-Value	*p*-Value	R^2^
Intercept	1.741	0.072	24.185	**<0.001**	-
Herbivory rate (%)	−0.063	0.021	−3.012	**0.002**	0.16

## Data Availability

All data will be available upon reasonable request.
